# University of Buffalo, Dental Department

**Published:** 1896-07

**Authors:** 


### Editorial, Etc.

### UNIVERSITY OF BUFFALO, DENTAL DEPARTMENT.

The fourth annual Commencement of the Dental Department of the University of Buffalo, was held in connection with the Department of Medicine and Pharmacy, in Music Hall, in the City of Buffalo, on the evening of Tuesday, May 5th, 1896.

The Oral examination before the Board of Curators was held during the day, and the members of the Senior Class who had passed the Faculty examinations were unanimously recommended to the Council of the University for the Degree of Doctor of Dental Surgery which was accordingly conferred by the Chancellor.

The number of matriculates for the Session was 186. The number of graduates was 36, as follows :

Charles Arthur Bean, New York; Charles K. Buell, New York; Edward W. Bridgman, New York; Sidney W. Bunting, Canada; John H. Cameron, New York; Victor A. Clapp, New York; Edmund T. Comstock, New York; Horace S. Cutler, New York; Charles S. Decker, New York; Wm. B. Dickson, Canada; Carl S. Eaton, New York; Chas. E. Featherstone, Canada; James H. Gillam, Canada; Thomas G. Gibson, Canada; Arthur Kidder, New York; Ralfe M. Harlan, California; Herbert J. Lyle, Canada; Frank G. Lugsdin, Canada; Wilhelm Muller, Germany; Emmet E. Mills, Pennsylvania; Eugene L. Martin, New York; Wesley A. Parish, New York; Paul B. H, Quedenfeldt, Germany; James M. Rainie, New York; Richard B. Redway, New York; Henry F. Squire, New York; Carl S. Starkweather, New York; Lewis P. Sauford, New York; Charles A. Stewart, New York; Walter S. Stevenson, New York; Peter A. Stadlinger, New York; J. G. Van Walkenburgh, New York; Henry D. Warner, New York; Harry C. Webb, New York; Earl J. Woodworth, New York; Douglas H. Young, New York.

